# Sensitivity to Communication Partners During Naturalistic AAC Conversations in Cantonese Chinese

**DOI:** 10.3389/fpsyg.2021.686657

**Published:** 2021-08-18

**Authors:** Yen Na Yum, Soby Ka Wing So, Rosanna Yuen-Yan Chan

**Affiliations:** ^1^Department of Special Education and Counselling, The Education University of Hong Kong, Hong Kong, China; ^2^SAHK, Hong Kong, China; ^3^Centre for Perceptual and Interactive Intelligence, The Chinese University of Hong Kong, Hong Kong, China; ^4^Department of Information Engineering, The Chinese University of Hong Kong, Hong Kong, China

**Keywords:** augmentative and alternative communication, Cantonese Chinese, cerebral palsy, communication partner, complex communication needs, linguistic analysis, symbol

## Abstract

Previous studies have shown that graphic-based augmentative and alternative communication (AAC) output tend to be short and simple in structure with non-canonical word order, and that AAC users may show differences when communicating with peers compared to professionals such as speech therapists (STs). However, there was a lack of report for graphic-based AAC in the Chinese context, and the effect of communication partners had not been investigated systematically. In this study with 34 AAC users and 10 STs, we reported common and distinct features of free conversations in Cantonese graphic-based AAC, relative to AAC in other languages. We also found that AAC users were sensitive to different types of communication partners. In particular, when conversing with peers, AAC users produced long messages with equal proportion of questions and responses, which suggested active and bi-directional exchanges. In conversations with STs, AAC users showed high diversity in expressive vocabulary, indicating access to more semantic concepts. Results suggested that the base language and the communication partner are both influential factors that should be considered in studies of graphic-based AAC. The mobile AAC system facilitated free conversations in users with complex communication needs, affording an additional channel for social participation.

## Introduction

Augmentative and alternative communication (AAC) systems are commonly used by people with complex communication needs (CCNs) to supplement verbal communication, or in some cases, substitute for oral language. Systems developed for various communities have features (e.g., electronic vs. non-electronic, text-based vs. image-based) that facilitate usage in different contexts. McNaughton and Light (2013) raised important ways that mobile technologies impact the current use and continued development of ACC, including generating greater acceptance in society and wider dissemination of services. In addition, mobile technology allow for new patterns of communication. As AAC mobile applications support network connectivity and interactions between two remote devices, users can converse with others in real-time to achieve a two-way communication which was infeasible before. EasyDial^TM^ is a first-of-its-kind cloud AAC system for people with CCN in Hong Kong that mimicked the mobile telephone functions, developed by the second and the third authors (Chan, 2014; Chan et al., 2016). In the pilot implementation of EasyDial^TM^, various user tests showed that while some specific concepts were missing from the symbol list, the app interface was convenient and friendly. The majority of participants mastered symbol selection and message composition within 10 min and had conversations with minimal assistance from others (Chan et al., 2016). The EasyDial^TM^ system has been launched to the public sector in Hong Kong in November, 2019 by SAHK, which is a rehabilitation service provider serving more than 15,000 families on an annual basis. This paper reported a linguistic analysis on AAC conversational data collected from the cloud server and outlined potential applications of the results.

As an alternative to oral language, AAC systems may undergo a simplification process to facilitate language unit selection and output. However, the degree of change depends on the specific needs of the target group. On one end of the spectrum, an AAC system may contain the same lexicon as oral language, augmenting it with word typing and electronic speech synthesis. Users may produce identical messages as normal speech, although typically at a much slower rate and with altered syntax and pragmatics compared to non-AAC counterparts (Friginal et al., 2013, 2016). This type of AAC mainly caters for users who have normal verbal or intellectual abilities, but with difficulties in speech production. On the other hand, an AAC system may be drastically simplified from the base language to cater for users with limited verbal, intellectual, or motor abilities. An example is the picture exchange communication system (PECS), which is characterized by a much reduced vocabulary representing core concepts with words and simple pictures. This type of AAC is suited for children or individuals with developmental disabilities, e.g., autism spectrum disorder (Chen et al., 2015; An et al., 2017), intellectual disabilities (ID) (Deckers et al., 2017), or cerebral palsy (Chan et al., 2016; Soto and Clarke, 2017, 2018).

In aided AAC systems such as PECS, selection and display of core vocabularies is highly specific to the language and needs of the target users. Many corpora had been developed with the intention to identify the optimal set of symbols for AAC. Speech corpora came from individuals with normal speech abilities [e.g., English-speaking school-age children in Boenisch and Soto (2015); Mandarin speaking adults in Chen et al. (2009) and Liu and Sloane (2006)] or from the target population [e.g., Dutch-speaking children with Down Syndrome in Deckers et al. (2017)], and text corpora were also considered (e.g., Mühlenbock and Lundälv, 2011). An efficient AAC system should provide concepts that match what the users need to convey. For example, an AAC system used in school setting will need academic concept representation. McCarthy et al. (2017) showed that many age-appropriate concept words were not adequately represented or easily accessible in four commercially available AAC systems. The authors highlighted the need for educators to recognize and address the limitations of basic concept content in pre-packaged AAC software or applications. In informal settings, conversations may exhibit a greater social orientation, with the main focus on daily life and common topics of interest. Free conversational contexts provide another setting to examine vocabulary selection and usage in an ecologically valid manner.

Many studies have shown that AAC vocabulary and morpho-syntax does not parallel speech or written language (Bernardi and Tuzzi, 2011; Friginal et al., 2013, 2016). Indeed, it has been argued that using AAC requires separate skills apart from those supporting oral language (Light, 1997; Smith, 2015). Although AAC comprehension and expression exhibits a wide range of grammatical complexities, in general, utterances tend to be shorter than would be expected based on participant profiles (Binger and Light, 2008). In a review of 31 studies pertaining to morphology and syntax in graphic symbol-based AAC, Smith (2015) reported four main linguistic patterns: (1) dominance of single-symbol output, (2) persistence of simple clause structures, (3) changes in word order, and (4) errors in inflectional morphology. As Chinese has flexible word order and minimal inflectional morphology, it contrasts with the bulk of the literature in English and European languages.

Conversation-based AAC intervention can increase communication abilities in target users, such as spontaneous communication and use of requests (Soto and Clarke, 2017, 2018). Individuals with CCN using aided AAC have been described as passive responders, as they tended to ask few questions and follow the set topics (e.g., Light, 1988; Clarke and Kirton, 2003). In a study with 12 children with physical disabilities using AAC systems with their speaking peers in school (Clarke and Kirton, 2003), children with CCN were significantly more likely to respond than initiate interactions compared to their naturally speaking peers. Even so, the distribution of turn taking in these conversations was more equal than what had been identified in conversations between adults and children using AAC systems. Lund and Light (2007) found that during interactions with their caregivers, more experienced AAC users fulfilled most of their obligatory turns and more than half of their non-obligatory turns, with a majority of participants able to approach reciprocity in turn taking. The communicative functions most frequently used by AAC users were confirmations/denials and provision of information. In some cases, the passiveness may be because the physical limitations of the user led to less control over the use of the AAC system. Pinto and Gardner (2014) reported that a child was able to use eye–gaze strategies to indicate interests both within and outside the AAC system, and the communication partner is tasked to be sensitive to these signals. Overall, individuals with CCN demonstrated ability to use AAC to serve a variety of communicative functions, but there seemed to be differences in usage under different contexts and with different communication partners (e.g., peers vs. caregivers vs. professionals).

Considering the diversity and impact of language type, user characteristics, and usage contexts on AAC output, a cross-language study may be of particular value in examining the universality of previous observations. This study reported Cantonese Chinese graphics-based AAC free conversations to examine how individuals with CCN use AAC with different communication partners, i.e., peers or speech therapists (STs). The usage pattern of STs toward individuals with CCN was also investigated as a comparison group. On the symbol level, we examined the core vocabulary used in free conversations, including frequency distribution and commonality of use in different groups. On the message-level, we compared message length, proportion of single symbols, type-token ratio, and communicative functions across groups. On the conversation level, we compared if the overall conversation length and the number of turns differed between user–user and user–ST conversations. We expected that individuals with CCN and STs would show different usage patterns that may reflect their verbal and motor abilities. The comparison between communication partners would show how social contexts influence language use in individuals with CCN. We also hypothesized that the data patterns would reveal some linguistic characteristics common to all AAC systems and some characteristics specific to Chinese.

## Materials and Methods

### Participants

The participants were 34 individuals (14 females, mean age = 32.1 years, SD = 15.4, range = 10–55) with CCNs (hereafter, users) and 10 STs. Cerebral palsy (CP) was the main clinical diagnosis of the users (17 dyskinetic CP, 6 spastic CP, 6 mixed type CP, and 5 other diagnoses, e.g., epilepsy), and 85% of users had comorbid ID, of which 13 had mild ID, 15 had moderate ID, and 1 had moderate to severe ID. As the majority of users were adults, their language levels were assessed by STs using criterion-referenced assessment with the Reynell Developmental Language Scales, corresponding to receptive language age (mean = 4.1, SD = 1.2, range = 2–5). All of the participants were exposed to AAC before the study. They were able to use communication book or board with photos or picture presentation. A subset of them (*n* = 9) were also able to type *via* computer or mobile phone but at significantly reduced accuracy and efficiency. The participants were assisted by STs who were working in either rehabilitation or school settings with at least 4 years of experiences in clinical practice. All STs were experienced in training people with CCNs to use AAC.

### The EasyDial^TM^ System

The current study was performed with the EasyDial^TM^ system. The system’s purpose is to provide bidirectional mobile phone-like communication services in form of real-time exchange of AAC picture symbols over the networks. When using the system, users are shown an interface and symbol selection is accompanied by text-to-speech output of Cantonese Chinese in an adult female voice [see details in Chan et al. (2016)]. Currently, there are a total number of 665 communication symbols available in the system; while these symbols are classified into 17 categories (e.g., people, food, activities) according to the semantic nature. There are two important features in the system:

• The capability of performing semantic recommendation of AAC communication symbols using a recurrent deep learning algorithm designed by the project team; which greatly shortened the symbol selection time in users with severe motor and cognitive limitations; and
• The curation of a growing volume of anonymized AAC usage data that has a great potential to inform evidence-based speech therapy practices through big data analytics.

Besides, other usability adaptation features such as personalized application client interface and touch screen dexterity settings are also available.

### Procedure

Data collection occurred in 2016 and 2019 during the development and prototyping stages of the system, respectively. Informed consent to participate in the study was obtained from all participants. Participants had unscripted free conversations using EasyDial^TM^ as AAC support, in either face-to-face conversations or AAC-based mobile phone calls. Free conversation here was defined as message production specifically for communication, without limit on conversation topic. Since EasyDial^TM^ supported 1-to-1 messaging, the messages produced were directed to a receiver and can be interpreted as intentional communication.

Although EasyDial^TM^ was also used by caregivers and acquaintances, only data from users and STs were included. About 40 messages contained repeated symbols that were caused by motor control of users and were excluded from analysis. The trimmed dataset included 1108 messages with 31.9% between users (UtoU, users *n* = 25), 33.9% from users to STs (UtoST, users *n* = 26), and 34.1% from STs to users (STtoU, STs *n* = 10).

### Data Analysis

On the symbol level, descriptive statistics of the dataset were reported in terms of the type and token frequencies of the selected symbols across groups. The frequency distribution of symbol use in the three groups was additionally examined by plotting the log of the frequency against the log of the rank. Commonality scores were calculated by counting the percentage of participants who used a particular symbol, e.g., commonality of “you” is 100% if all participants have used it at least once in their messages. This measure complements lexical frequency in that high commonality shows that a symbol is widely used, not only repeatedly used by a select few participants.

On the message level, message lengths across groups were compared using linear mixed-effects models (Baayen et al., 2008) using the Satterthwaite method for degrees of freedom. A by-participant random intercept was used in the model to account for clustering of paired participants in UtoU and UtoST conditions and to minimize the effect of unequal number of observations from participants. *Post hoc* tests were run with Bonferroni adjustments for multiple comparisons. Chi-square tests were used to determine group differences in count data, including number of single symbol messages, number of repairs, and distribution of communicative functions. *Post hoc* comparisons between groups were done with Bonferroni adjustments for multiple comparisons. Two independent raters judged the communicative functions of the messages (inter-rater agreement = 94.6%). The disagreements contained cases where the intended meaning was ambiguous, for example, “you; eat” may be a directive, a statement, or a question lacking the question marker; “I; greet” may be a direct greeting or a comment on a past action; or a single message may be split into two: (1) “you; happy” + (2) “question marker.” Disagreements were resolved after discussion, prioritizing contextual clues from the conversations.

On the conversation level, the number of messages and turns within a conversation and their ratio were examined. A conversation was either defined by greetings at the beginning or end, or a separation in time. The messages were included only if the sender and receiver each had at least one turn in the conversation. Eight messages did not meet this criterion and were excluded from this analysis (0.008%). The remaining data were log-transformed to approximate a normal distribution being before submitted to linear mixed models with by-pair random intercept to compare user–user and user–ST conversations.

The mixed-effects models were run using GAMLj (Gallucci, 2019) on Jamovi (2020) on the R Core Team (2019). For all inferential statistical tests, α level was set at 0.05.

## Results

### Symbol Level

**FIGURE 1:**
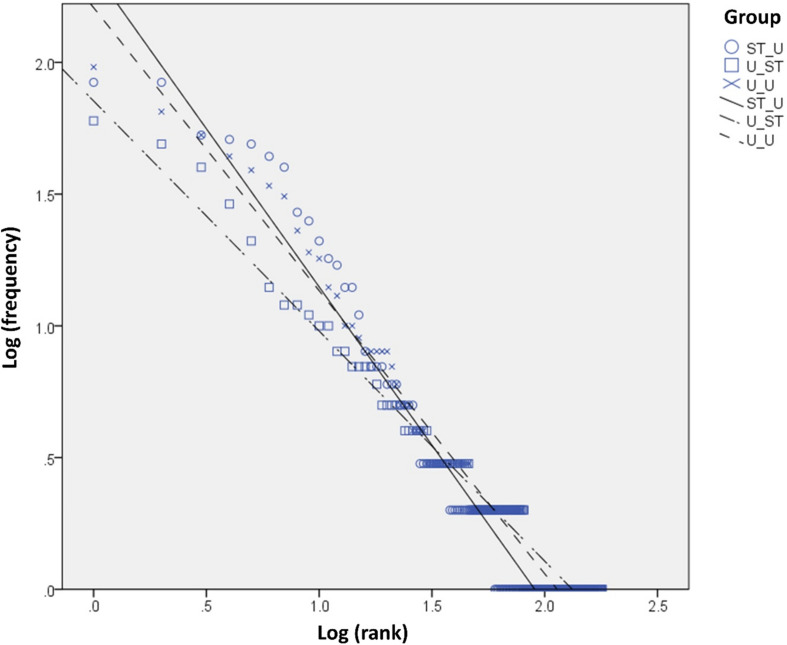
Log–log plot by participant group.

In the dataset, 2080 symbols were used (token counts), of which 290 were distinct (type counts). Overall, 182 symbols (type) appeared only once or twice in the corpus, so the core vocabulary used in free conversations is small. The 9 most frequently used symbols already accounted for over 50% of all symbols used, and 40 symbols represented 75% of total usage. The distribution of symbol use followed the Zipf’s scaling law [*P*(*r*) ∼ *r*^–α^] characteristic of natural languages, with *R*^2^ above 95% in all three sub-groups (Figure 1). Due to the limited size of the dataset, we did not conduct further statistical tests for this measure. In terms of parts of speech, nouns and verbs were the most frequently used and accounted for 23.1 and 22.4% of the symbols. Pronouns and interjections occurred at the next highest frequency at 19.8 and 19.3%, respectively. Adjectives (6.5%) and adverbs (1.6%) occurred with low frequency, while particles (2.7%), conjunctions (0.1%), and prepositions (0%) were used minimally. Symbols representing phrases with two or more words (e.g., take off jacket, play on the computer) were used 4.4% overall (see Table 1 for properties of symbols with the highest frequency, including the category and parts of speech).

**TABLE 1:** Features of the most frequently used symbols in the dataset.

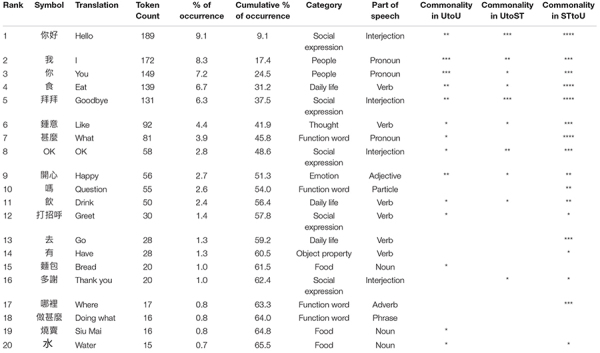

****81–100%; ***61–80%; **41–60%; *21–40%.

Among the high frequency symbols, commonality was highest in the STtoU messages, suggesting uniform word choice across different STs, while commonality was lowest in UtoST messages, indicating idiosyncratic word usage. The breakdown of type and token frequencies across groups can be found in Table 2. While the three sub-groups had comparable values for number of messages, the type count was higher and token count was lower for the UtoST group relative to the other two groups.

**TABLE 2:** Message characteristics by participant group.

Participant group	No. of message	Type count	Token count	Message length		% single symbol	% repairs
				Mean	SD		
Users to users	354	144	741	2.09	1.24	**46.3**	10.7
Users to speech therapists	376	180	581	1.54	1.05	**66.5**	**17.6**
Speech therapists to users	378	122	757	2.01	1.22	50.5	10.1
Overall	1108	290	2079	1.88	1.19	54.6	12.8

*Values in bold denote significant differences from expected values.*

### Message Level

The random effect likelihood ratio test suggested clustering of data by participant (*p* < 0.001), but the proportion of variance explained by the random effect was not high, with intraclass correlation (ICC) = 0.071. Results showed a statistically significant group effect *F*(2, 52.1) = 13.6, *p* < 0.001. Pairwise comparisons with Bonferroni adjustments indicated that the UtoST messages were significantly shorter than STtoU (*p* = 0.002) and UtoU (*p* < 0.001) messages, while the latter two groups did not differ from each other (*p* = 1.00; observed values in Table 2). A Chi-square test showed that there was group difference in the proportion of single symbol messages, χ^2^ (2) = 33.7, *p* < 0.001. *Post hoc* tests revealed that UtoU messages had significantly less single symbol messages, UtoST messages had significantly more single symbol messages, while STtoU messages did not differ from the expected values. There was also group difference in the proportion of repairs, χ^2^ (2) = 11.5, *p* = 0.003, where the UtoST messages had significantly more repairs than expected. Tabulated counts of four main communicative functions (i.e., convention, statement, question, and response), and an unspecified category are presented in Table 3. The Chi-square test indicated that communicative functions were different depending on group, χ^2^ (8) = 211, *p* < 0.001. The most frequent function was conventionalized social language (e.g., greetings, thank you), which occurred significantly more often in STtoU messages and less often in UtoU messages. The next frequent function was statement, which included sharing new information or commenting, and STtoU messages had significantly less of this function than expected. Questions and responses occurred similar numbers of times overall, suggesting appropriate social responsiveness. As predicted, these functions showed a clear difference in ST and user interactions, where STs tended to ask questions rather than answer them, while users tended to answer questions but not raise them. Remarkably, users had a balanced question and answer proportion when talking amongst themselves. The unspecified category included null messages, consecutive repeated messages, and messages with unclear meanings. There are more unspecified UtoU messages and less unspecified STtoU messages than expected. The request function was notably absent in this dataset, likely due to the conversation context where the focus was casual social exchange rather than training for AAC utility.

**TABLE 3:** Contingency table of communicative functions by participant group.

		Communicative Functions					
Groups		Convention	Statement	Question	Response	Unspecified	Total
Users to users	Observations	**89**	120	59	58	**28**	354
	% within row	**25.1**	33.9	16.7	16.4	**7.9**	100.0
Users to speech therapists	Observed	106	109	**18**	**125**	18	376
	% within row	28.2	29.0	**4.8**	**33.2**	4.8	100.0
Speech therapists to users	Observed	**135**	**48**	**143**	**46**	**6**	378
	% within row	**35.7**	**12.7**	**37.8**	**12.2**	**1.6**	100.0
Overall	Observed	330	277	220	229	52	1108
	% within row	29.8	25.0	19.9	20.7	4.7	100.0

*Values in bold denote significant differences from expected values.*

### Conversation Level

There were 35 conversations among 21 user–user pairs and 58 conversations among 29 user–ST pairs. The mean number of messages within a user–user conversation was 9.94 (SD = 12.1, range = 2–68), while that for user–ST conversation was 13.0 (SD = 7.68, range = 4–52). The random effect likelihood ratio test indicated significant clustering of data by different conversation pairs (*p* = 0.009), with ICC of 0.541 showing that pair-specific differences explained much of the variance in the data. Still, user–user conversations were statistically shorter than user–ST conversations, *F*(1, 35.9) = 4.14, *p* = 0.049. Likewise, the mean number of turns within a user–user conversation was 6.74 (SD = 5.93, range = 2–25), less than that for user–ST conversations, which was 9.84 turns (SD = 4.19, range = 2–26). The random effect likelihood ratio test for the pair random intercept was marginally significant (*p* = 0.095), with ICC of 0.471. The difference in number of turns between groups was also statistically significant, *F*(1, 30.4) = 8.37, *p* = 0.007. The ratio of number of turns to number of messages was 0.774 (SD = 0.201) and 0.791 (SD = 0.124) for user–user and user–ST conversations, respectively. The random effect of pairs was significant (*p* = 0.017), ICC = 0.309, however, there was no statistical difference between groups for this metric, *F*(1, 38.6) = 1.49, *p* = 0.230.

## Discussion

In the overall type count of the current dataset, less than half of the available symbols in EasyDial^TM^ were used. Despite clear differences in the linguistic features of graphic symbol-based AAC and the full Cantonese language, the frequency distribution of AAC data in free conversations in the three sub-groups all exhibited the Zipf’s scaling law. The Zipf’s law has been reported in many natural languages, including in specific populations such as child (e.g., Baixeries et al., 2013) or elderly language use (Abe and Otake-Matsuura, 2021). As seen from Figure 1, the exponent α is close to 1 for all three groups, consistent with these studies. Interestingly, Baixeries et al. (2013) observed that syntactic complexity measured by mean length of utterance is negatively related to the exponent values in child language development, but Abe and Otake-Matsuura (2021) found no relation between cognitive functions and the exponent value in free conversations in older adults. Although the present dataset was not of sufficient size to do more fine-grained analyses, future studies could explore such relationships with AAC user characteristics.

Cantonese graphic symbol-based AAC elicited distinctive morpho-syntactic usage patterns that aligned with previous reports in other AAC languages, specifically, single symbol use and simple clause structure (Smith, 2005; Binger and Light, 2008). The high proportion of single symbol utterances were partially due to inclusion of greetings common in phone calls (e.g., “hello,” “goodbye”), and simple answers to questions. This is because even simple questions need several symbols while answers can be single symbol (e.g., “you like drawing?” vs. “yes”). Another reason is that symbol selection and output was usually effortful in AAC users, and there could be difficulty in motor control or command of the system for users in conversations, as evidenced by the number of repairs and messages with unspecified functions. So the prevalence of single symbol may be a strategic choice to maximize communication efficiency. Similarly, simple clause structure may be adopted to convey the central meaning of messages, with omission of function words or less important elements such as adjectives and adverbs. Overall, there were not many multi-symbol messages, and the symbol set included some common phrases to enhance communication efficiency, so coding of grammatical structure of individual messages was not done. Nevertheless, canonical SVO word order was observed for many messages with multiple symbols, and errors in word order were not particularly noted (unlike e.g., Binger and Light, 2008). However, since Chinese sentences may have a topic-comment structure and word order is not strict, even if symbols did not follow a typical word order, the message could still be interpreted – only about 5% messages had unspecified communicative function. Therefore, in terms of lexical choice and dominance of single symbols, we did not note much cross-language differences in AAC usage. Meanwhile, because of the flexible word order and minimal morphological inflections that is characteristic of Cantonese Chinese, grammatical errors in word order or verb or number agreements did not occur, and message meanings were largely interpretable in context. In a small number of messages, errors appeared to be driven by picture processing. For example, a student used the symbol for “father” when addressing the ST, presumably because the picture depicted an older male. This suggested that picture representations in the AAC system is important for its appropriate use regardless of the verbal language.

A salient finding was the systematic differences in message construction in how users with CCN and professionals such as STs used AAC. Kent-Walsh et al. (2015) had reported in a meta-analysis that interventions by AAC partner instruction were highly effective across a range of participant types, intervention approaches, and outcome measure characteristics. In AAC interventions, modeling, expectant delay, and open-ended question asking were frequently targeted interaction skills. Although the current data were collected under free conversation instead of explicit intervention context, STs still employed a structured client-centered approach and asked many questions to stimulate responses from users with CCN. Results from the commonality scores and analysis of communicative functions supported this interpretation. In general, users appeared more reserved when talking to STs compared to peers, producing shorter messages with more repairs. This was likely because they were fulfilling their obligatory turns by responding to questions from STs. Users might also be sensitive of the usual social roles, i.e., that the STs were the “teachers” and they were the “students,” and so they adopted a more receptive mode of communication. On the other hand, users produced a wider range of vocabularies when talking to STs compared to their peers, as indicated in the type count and the commonality scores. This suggested that even free conversations with STs may promote the diversity of expressive vocabulary in users. When users conversed with other users in free conversations, they initiated questions more often and produced more tokens than when they conversed with STs. They also used less conventions, indicating a more casual register. This pattern suggested that EasyDial^TM^ may facilitate active and bidirectional pattern of communication with peers (cf. Clarke and Kirton, 2003), in line with professionals’ views that smart phone texting with picture symbols and speech can increase independence and participation in users with CCN (Buchholz et al., 2013). In terms of the overall quality of conversations, user–ST conversations were longer than user–user conversations with more messages and turns per conversation. However, reciprocity of conversation partners as indexed by turn-taking behavior did not differ between user–user and user–ST conversations.

In sum, this study reported linguistic analyses of graphic symbol-based AAC usage in a sample of users with CCN using Cantonese Chinese, with similarities and differences with AAC in other languages. We found that users with CCN had different usage patterns when conversing with peers and STs, suggesting sensitivity to communication partners or conversation topics, but both contexts could be valuable to their social communication. A limitation in this study is that we did not explore the effects of face-to-face vs. remote messaging, although this could yield differences in the choice of symbols and the contextual understanding. Further studies could address this question in light of the social distancing measures, which could restrict in-person communications. Although the usage data in the current report are limited in size compared to typical language corpora because of the nature of AAC, anonymized AAC usage data are continually accumulated as EasyDial^TM^ is used in the local community. Availability of these data in the future could allow for further cross-linguistic comparisons between AAC in Chinese and other languages. Our data can be used to deduce the communicative needs of the diverse and understudied population of people with CCN. Results will inform future enhancement of EasyDial^TM^ as well as other AAC systems, thereby improving service provision and ultimately equal access and social inclusion.

## Data Availability Statement

The original contributions presented in the study are included in the article/Supplementary Material, further inquiries can be directed to the corresponding author/s.

## Ethics Statement

The studies involving human participants were reviewed and approved by SAHK. Written informed consent to participate in this study was provided by the participants’ legal guardian/next of kin.

## Author Contributions

YY contributed to the analysis of the results and to the writing of the manuscript. SS and RC designed the EasyDial^TM^ system and performed the user study. All authors contributed to the article and approved the submitted version.

## Conflict of Interest

The authors declare that this study was partially supported by the Centre for Perceptual and Interactive Intelligence (CPII) Ltd. under the Innovation and Technology Fund. The funder was not involved in the study design, collection, analysis, interpretation of data, the writing of this article or the decision to submit it for publication.

## Publisher’s Note

All claims expressed in this article are solely those of the authors and do not necessarily represent those of their affiliated organizations, or those of the publisher, the editors and the reviewers. Any product that may be evaluated in this article, or claim that may be made by its manufacturer, is not guaranteed or endorsed by the publisher.
